# Vegetarian diets in childhood and adolescence: Position paper of the nutrition committee, German Society for Paediatric and Adolescent Medicine (DGKJ)

**DOI:** 10.1186/s40348-019-0091-z

**Published:** 2019-11-12

**Authors:** Silvia Rudloff, Christoph Bührer, Frank Jochum, Thomas Kauth, Mathilde Kersting, Antje Körner, Berthold Koletzko, Walter Mihatsch, Christine Prell, Thomas Reinehr, Klaus-Peter Zimmer

**Affiliations:** 1 Children’s Hospital, University of Giessen, Giessen, Germany; 2 Neonatology, Charité Universitätsmedizin Berlin, Berlin, Germany; 3 Evangelisches Waldkrankenhaus Berlin Spandau, Berlin, Germany; 4 Practice for Paediatrics and Adolescent Medicine/Sports and Nutrition Medicine Ludwigsburg, Committee for Prevention and Early Therapy BVKJ Cologne, Cologne, Germany; 5 Research Department of Child Nutrition, Department of Paediatrics, Ruhr-University Bochum, Bochum, Germany; 6 Paediatric Research Center, Dept. Womens’ and Childrens’ Medicine, Univ. Leipzig, Leipzig, Germany; 7 LMU – Ludwig-Maximilians-Univ. Munich, Dr. von Hauner Children’s Hospital, Munich, Germany; 8 Nutrition Committee of the German Society for Paediatric and Adolescent Medicine (DGKJ), Chausseestr, 128-129 Berlin, Germany; 9 Children’s Hospital, Heliosklinikum Pforzheim, Pforzheim, Germany; 10 Department of Pediatric Endocrinology, Diabetology and Nutritional Medicine, Vestische Children’s Hospital Datteln, University Witten/Herdecke, Witten, Herdecke Germany

**Keywords:** Omega-3 fatty acids, Iron, Vitamin B12, Malnutrition, Bioavailability

## Abstract

In Western countries, vegetarian diets are associated with lower intakes of energy, saturated fatty acids and animal protein and higher intakes of fibre and phytochemicals, compared to omnivorous diets. Whether the corresponding health benefits in vegetarians outweigh the risks of nutrient deficiencies has not been fully clarified. It should be noted that vegetarians often have a higher socioeconomic status, follow a more health-conscious lifestyle with higher physical activity, and refrain from smoking more often than non-vegetarians. The nutritional needs of growing children and adolescents can generally be met through a balanced, vegetable-based diet; however, due to their higher nutrient requirements per kilogramme of body weight, vegetarian children have a higher risk for developing nutrient deficiencies than adults. With a vegetarian diet, the mean intakes of some nutrients, such as the omega-3 fatty acid docosahexaenoic acid (DHA), are lower than in omnivores or those eating fish. For other nutrients, such as iron and zinc, the bioavailability from vegetable foodstuffs is reduced when the intake of phytates and fibre is high; thus, the prevalence of iron deficiency can be increased despite high vitamin C intake. In addition, vitamin B12 is only found in animal-source foods. Vitamin B12 should be supplemented in people of all age groups who follow a strict vegan diet without consuming animal products. A vegetarian diet in childhood and adolescence requires good information and supervision by a paediatrician, if necessary, in cooperation with an appropriately trained dietary specialist.

## Introduction

Paediatricians currently encounter a growing number of families who opt for a vegetarian diet. They are confronted with numerous health-related questions as well as the need for medical supervision and care. Current guidelines for routine paediatric care in Germany stipulate inclusion of preventive nutrition counselling [6]. Therefore, the Nutrition Commission of the German Society for Child and Adolescent Medicine comments here on vegetarian diets in childhood and adolescence.

Over the last few years, there appears to be an increased interest in vegetarian diets in Germany, although robust prevalence data are lacking. According to estimates from the German Vegetarian Association (VEBU), the number of vegetarians has increased from 7% to 10% during the last 10 years, while the number of vegans is estimated to be around 1% [44]. The results of a national, representative survey on health in Germany (DEGS1, 2008–2011) revealed that 4.3% of the adult population is vegetarian; the highest proportion of vegetarianism was found among young adults ages 18–29 (9% of females, 5% of males) [26]. Vegetarianism is far less common among children and adolescents. Among 14 to 17 year olds who participated in the 2003–2006 KiGGS survey (a national survey on the health of children and adolescents in Germany), 2% of male adolescents and 6% of female adolescents considered themselves vegetarian [25].

The variation in these estimates may be due to an overlap of types of vegetarianism and lack of standardization of terminology. In Germany, there are no uniform definitions for the terms “vegetarian” and “vegan” (Table 1). In surveys about types of vegetarianism, participants frequently define their diet type differently from established types of vegetarianism [26].

**Table 1:** Characteristics of a vegetarian diet (modified according to Mensink et al. [26] and Kersting [16])

Type of diet	Excluded food groups	Reduced nutrient intake
Lacto-ovo-vegetarian	Meat, fish products	Meat: vitamin B12, animal protein, iron, zinc (high bioavailability) Fish: iodine, omega-3 fatty acid, e.g. DHA
Lacto-vegetarian	Meat, fish products, eggs	Same as above Eggs: animal protein, vitamin D and A
Ovo-vegetarian	Meat, fish products, milk and milk products	Same as above Milk: animal protein, calcium, iodine, vitamin B12, B2, D, A
Pesco-vegetarian	Meat products	Same as above but with fish
Flexitarian (generally vegetarian)	Meat and fish products, occasional consumption of small portions	Same as above Only slight reduction in nutrients
Vegan	All animal-based products (meat, fish, milk, eggs, honey)	Same as above In addition, primarily vitamin B12
Raw vegans	All animal-based products, certain plants, cooked foods	Same as above In addition, calories and fat

*DHA* docosahexaenoic acid

### Characteristics of vegetarian diets

Assessing vegetarian diets specifically for children and adolescents can be complicated for a number of reasons. First and foremost, there is a variety of vegetarian diet types which exclude certain foods and can therefore contribute to insufficient intake of particular nutrients (Table 1). In some diets, nutrient intake can be dramatically reduced, e.g. the lack of vitamin B12 in a diet as a result of eliminating animal-based foodstuffs.

A lacto-ovo-vegetarian diet, which comprises milk and eggs supplying key nutrients but not meat or fish, is the most common type of vegetarianism. Strict vegetarianism (veganism) excludes all foods sourced from animals. In general, the risk of insufficient nutrient intake rises with increasing degree of dietary restrictions [33]. With vegan diets, for example, this risk rises if certain plant-based foods such as legumes are avoided. Furthermore, children may have preferences for and aversions to certain foods, which can contribute to an increased risk of undernutrition.

### Health implications of vegetarianism among children and adolescents

Few studies explored alternative diets and their implications for children, and those available usually are based on small sample sizes. Many of these studies were performed in the 1980s/1990s, which means that present-day food selection and habits are not reflected. A recent systematic review was published which assessed several characteristics of the types of vegetarianism in paediatric populations, including data on diet, nutrition and health status. Of the 24 publications from 16 studies in industrialized countries, the average number of children and/or adolescents per age group was only 35. One publication was performed in Germany but most were carried out in the USA and Poland; some studies also were performed in Great Britain, Slovakia and Belgium.

There are a number of reasons why a vegetarian diet may be preferred. Most often, the reasons are rooted in ethics, moral, religious or environmental viewpoints. This can include being opposed to the manner of food production, e.g. intensive farming (so-called factory farming) [26]. In the past, a vegetarian diet was associated with an appreciation of “natural” foods, i.e. foods with a low degree of processing [19]. More recently, the growing demand for meat alternatives and milk alternatives has led to a sharp increase in sales of processed vegetarian and vegan foods. From 2010 to 2015, the average annual growth in sales of vegetarian products was 17% [14]. These foods are produced in a similar manner to conventional processed foods with high levels of processing, and often high contents of sugar, saturated fat and additives whose physiological nutritional value is seen critically. On the other hand, these foods may contribute to intakes of critical nutrients, depending on fortification [35]. As these ready-made vegetarian products are in ever greater demand, it is nearly impossible to apply the results from earlier studies on vegetarian diets to the current situation.

Furthermore, vegetarian diets are often associated with other lifestyle characteristics, presenting a challenge for observational studies to capture the effects of a vegetarian diet separately from other lifestyle choices. Controlled intervention trials with long follow-up are difficult to perform in children. Conclusions from existing studies are often influenced by selection bias, that is, the choice of the study sample may be dependent upon existing eating habits. Frequently, recruitment for such studies may take place in contexts in which a certain health-consciousness is present (e.g. Seventh-day Adventists schools), and therefore, the results may be influenced by other lifestyle factors. This is applicable for two of the studies with children eating vegan diets, which were performed in the 1980s [29, 38]. The families participating in the study had a high socioeconomic status, the children received nutritional supplements or enriched food products, and there was no control group. Other challenges include the lack of recency of the performed studies, the sample sizes for the various types of vegetarianism (Table 1), and a lack of quantitative data and quality assessment of the studies. Considering the growing number of vegetarian families and the limited evidence base, robust studies which assess vegetarian diets among children are needed. These studies should consider that the socioeconomic status of vegetarians is higher than that of non-vegetarians [26].

With a careful interpretation of the existing studies, one can conclude that the risk of nutrient deficiency in childhood grows with an increasing level of dietary restrictions. Infants after the end of the period of full breastfeeding and young children are at greatest risk [16, 17, 33, 39].

### Growth and physical development

A recent review [39] summarizing studies in vegetarian vs. omnivorous diets in children [2, 3, 4, 9, 10, 22, 28, 32, 36, 40] found that physical development between the two groups of same-aged children was generally similar in terms of height, weight and body mass index (BMI). However, some studies from the 1980s and 1990s [11, 23, 38] report a reduced albeit still normal body weight and a lower body fat mass in children on vegetarian diets. In a study of 11- to 14-year-old school-age children, the average weight of the children with vegetarian diets was 4 kg lower than of those with omnivorous diets [23]. A study in Flemish children and adolescents aged 6–17 years reported a lower energy intake compared to reference data, but only adolescents ages 10–17 years were smaller, weighed less and had lower BMI [11]. Children with vegetarian diets performed worse in strength tests but better in endurance tests compared to the reference population. In a review, Sabaté and Wien [37] reported that vegetarian children are thinner than omnivorous children, with the BMI discrepancy becoming more pronounced during adolescence. Studies in adults show that plant-based diets have a lower caloric density and higher fibre content than omnivorous diets; at the same time, vegetarians consume less sweets or foods with added fats [31]. Studies with children provide only limited reliable data on caloric, protein and fat intakes, yet they suggest a lower intake of total calories, protein and total fat in vegetarian diets [11, 32, 38], whereas other studies showed no difference. It appears that normal weight and height development is achievable with a balanced vegetarian diet that includes some animal-source foods [7, 17].

### Supply of critical nutrients

Individuals may be at risk of an inadequate nutrient supply if nutrients

1. are only found in foods sourced from animals, e.g. vitamin B12,
2. can only be found in small amounts in plant-based foods, e.g. calcium, and
3. may not be absorbed well due to the high content of inhibitory substances like phytates and oxalates in plant-based foods (whole grain, legumes and spinach), e.g. iron and zinc.

The risk may be further elevated if individuals are not willing to seek out professional dietary counselling and/or take supplements or fortified foods [43].

Dietary proteins are divided into two categories based on their biological value—those that are sourced from animals and those that are sourced from plants. In research studies on total protein intake, children with vegetarian diets attained levels of protein as the reference group, i.e. their total protein intakes did not differ from those children with omnivorous diets [1, 24]. Krajcovicová-Kudlácková et al. [23] found that children with vegetarian diets consumed less milk and milk products than non-vegetarians—suggesting an increased risk of insufficient protein supply. Considering the limited data from nutrition surveys, it cannot be assumed that this is related to the amount of protein consumed. Protein needs can easily be met with a lacto-ovo-vegetarian diet; even if animal-based products are excluded, these needs can be met, after infancy, through a combination of grains and legumes. During infancy and in later years, a soy-based infant formula can provide an additional source of nutrients.

Total protein and albumin concentrations can be measured in blood serum. However, it should be noted that a reduction in overall protein levels is not an early indicator of protein deficiency and can have other non-diet related causes.

Iron is an important nutrient particularly for infants older than 6 months, young children, and menstruating adolescents. The bioavailability of iron in plant-based foods (Fe^3+^) is lower compared to heme iron from meat (absorption 2–5% vs. ca. 20% in heme iron). The data regarding higher incidence rates of iron deficiency or iron deficiency anaemia in children with vegetarian diets are inconclusive. In two published studies with small sample sizes of infants and children up to age 2, there was no indication of increased iron deficiency anaemia (as measured by haemoglobin [40, 42]). Some studies in older children demonstrated higher rates of iron deficiency among vegetarians vs. omnivores [23, 28]; others however have found no significant difference in iron levels. However, the total iron intake was higher in vegetarians than in omnivores [32, 38]. Whether the lower bioavailability of iron from plants can be offset through a simultaneous increase in vitamin C is questionable since iron deficiency was still more common in children with vegetarian diets vs. omnivorous diets, even when iron intakes were comparable and vegetarian children had significantly higher vitamin C intakes [10].

During physical exams, paediatricians should be attentive to signs of anaemia (pale skin, conjunctiva, and fingertips and tachycardia). Diagnostic parameters for iron deficiency are microcytic cells on blood tests (mean corpuscular volume, MCV < 74 fl) combined with low ferritin levels. Microcytic anaemia can be distinguished from other forms of anaemia with a high red cell distribution index (RDI) or red cell distribution width (RDW) which is higher with iron deficiency anaemia (> 15%). In addition, in the case of manifested anaemia, there may be lower transferrin saturation and a relative reticulocytopenia, as well as a high concentration of soluble transferrin receptors (this is however also high with some chronic illnesses or haemolytic anaemia). Therefore, measuring iron levels alone has no diagnostic significance.

Adequate iodine intake is essential for optimal physical and neurological development for children and adolescents. For children, important sources of iodine, in addition to iodized salt, are milk products [15]. The risk for iodine deficiency increased if relevant sources of iodine supply, such as fish, meat, eggs and milk, are removed from the diet [27], which needs to be compensated for by preferential selection of iodine-enriched foods such as bread and iodized table salt.

The gold standard for assessing iodine status is to evaluate iodine excretion in a 24-h urine collection. Epidemiological studies sometimes measured a urinary iodine concentration related to creatinine in spot urine samples. However, it is important to note that creatinine reference values are dependent on both age and development [41]. Inductively coupled plasma (ICP) mass spectrometry is the gold standard for measuring these trace elements, yet it is expensive and is not available in all diagnostic laboratories. There are a number of reference values provided by the World Health Organization (WHO) and by German studies, although the cut-offs vary substantially, making it difficult to draw definitive conclusions [8]. Since iodine is necessary for thyroid hormone synthesis, it is also recommended to test thyroid parameters. However, a high concentration of thyroid-stimulating hormone (TSH) and normal to low thyroxin (T4) levels with normal triiodothyronine (T3) levels can be an indicator (but does not have to be). This constellation of values must be differentiated from other forms of hypothyroidism, in particular autoimmune thyroiditis and isolated TSH elevation, which can be common in obese children.

Vitamin B12 is missing from a vegan diet, since it is only provided by animal-based (or fortified) products. Studies have shown that children with vegan diets may compensate this through taking supplements or through consumption of fortified foods such as fortified soy drinks [29, 30]. Vitamin B12 provided in certain foodstuffs such as fermented soy milk, mushrooms or algae do not adequately meet children’s vitamin B12 needs due to poor bioavailability [46]. Depending on a person’s vitamin B12 status and age, oral supplements of 5–25 μg/day are recommended; the recommendations of 1–3 micrograms/day, given in the D-A-CH reference values pertain only to individuals who have sufficient reserves [8]. In the case of manifested vitamin B12 deficiency, paediatricians are advised to treat initially with a single, one-time intramuscular injection of e.g. 1000 μg.

In diagnostic testing, low levels of holotranscobalamin in the blood or urine can indicate a deficiency and depletion of vitamin B12 reserves. This lab result provides the earliest indication of a vitamin B12 deficiency, even in cases where clinical and haematological symptoms are not yet apparent. An elevated level of methylmalonic acid (MMA), typically along with high homocysteine levels, points to a functional vitamin B12 deficiency. This diagnostic test can be expensive, and its diagnostic value is debated. Measuring vitamin B12 levels alone usually does not establish the diagnosis of deficiency. Since a vitamin B12 deficiency can lead to a macrocytic anaemia, it is recommended to perform a blood test. It should be noted that a vitamin B12 deficiency is often concurrent with an iron deficiency.

Calcium and vitamin D play a decisive role for bone health. According to data from Sanders [38] and Ambroszkiewicz et al. [3], calcium intake was approximately 50% lower in children with a vegetarian diet vs. those with an omnivorous diet. Vitamin D levels are also lower in vegetarians [3]. While the majority of vitamin D dietary requirements can be met through endogenous synthesis, there is a considerable risk of vitamin D deficiency in children and young adults. This is particularly relevant for children with vegetarian diets, as negative associations were observed in relation to markers of bone metabolism [3].

The gold standard for vitamin D (25-OH-Cholecalciferol) measurement is high-performance liquid chromatography (HPLC) or liquid chromatography-mass spectrometry (LC/MS), but not through enzyme-linked immunosorbent assay (ELISA) which was performed in many studies. Until today, there is no internationally accepted standard or reference value for vitamin D tests. It is not necessary to measure calcitriol (1–25 dihydroxy-cholecalciferol) values. Vitamin D is unstable under light so serum tubes should be protected from light during storage and transported in order to prevent false-positive results. In the case of manifested vitamin D deficiency, high serum/plasma concentrates of parathyroid hormones and alkaline phosphatase are expected.

Although vegetarian diets typically have higher consumption of polyunsaturated fatty acids (PUFA) from plant-based oils than do omnivorous diets, the consumption of long-chain omega-3 fatty acids (particularly docosahexaenoic acid (DHA)) frequently is below recommended intakes. Krajcovicová-Kudlácková et al. [22] showed that consumption of omega-3 fatty acids was lowest among vegans and highest among pescatarians. Evidence regarding the need for long-chain omega-3 supplements after infancy is still debated, despite the fact that some studies that demonstrate an influence on neurological function in childhood [5, 30].

### Vegetarianism during pregnancy and breastfeeding and its effect on infants

Numerous studies showed that breastfeeding infants of mothers with vegan diets can develop severe vitamin B12 deficiency and irreversible neurological damage [13, 18, 34, 45]. Thus, mothers with vegan or vegetarian diets need to take vitamin B12 supplements, preferably combined with other critical nutrients such as iron, zinc, iodine, vitamin D and DHA [20, 21].

In the absence of fish, individuals can acquire DHA through plant-based sources like DHA-rich oils from the microalgae *Uklenia* and *Schizochtrium*. In the European Union (EU), these foods are considered “novel foods” and are permitted as nutrition supplements. Even with mixed lacto-ovo-vegetarian diets, vitamin B12 and other nutrients may be lacking [12]. All pregnant and breastfeeding mothers with vegan and vegetarian diets should seek medical consultation and should be evaluated for possible deficiencies in critical micronutrients (e.g. through blood tests with evaluation of vitamin B12, holotranscobalamin, ferritin, vitamin D, zinc, iodine and MMA in the urine).

## Conclusions and recommendations

– A balanced, omnivorous diet with ample consumption of plant-based foods and moderate consumption of meat, fish and milk products is the recommended diet for children because nutrient requirements are most easily and most likely met.

– Restrictive diets are associated with an increased risk of nutrient deficiency: the stricter the diet, the greater the risk.

– A balanced, lacto-ovo-vegetarian diet as part of a healthy lifestyle during infancy, childhood and adolescence can meet nutritional requirements, support normal growth and age-appropriate development. However, special attention should be given to ensure higher iron intakes in order to compensate for lower iron bioavailability.

– A vegan diet (without any animal-derived foods) over an extended period of time regularly leads to vitamin B12 deficiency if the diet is not appropriately supplemented. Providers should pay attention to the intake and status of iron, zinc, iodine, DHA, calcium, protein and calories in order to prevent serious clinical complications such as growth faltering, anaemia or neurological damage.

– Vitamin B12 is not only essential for children and adolescents with vegan diets, it is also critical during pregnancy and breastfeeding. Insufficient vitamin B12 can result in severe deficiency symptoms in infants and young children. Pregnant and nursing mothers with vegan diets should take vitamin B12 through oral supplements to meet nutritional requirements.

– Nutrition and diet should be a part of paediatric care and prevention. Paediatricians caring for children with vegetarian or restrictive diets should monitor physical development and dietary intakes, if necessary, in cooperation with an appropriately trained dietician/nutritionist. Blood tests may be necessary in certain cases to assess nutrient status. Additional foods as well as supplements should be recommended in the case of inadequate nutrient intakes or nutrient deficiencies.

## Data Availability

See references.

## Abbreviations

1–25 dihydroxy-cholecalciferol: Calcitriol
25-OH-Cholecalciferol: Vitamin D
BMI: Body mass index
DGKJ: German Association for Peadiatrics and Adoloscent Medicine
DHA: Docosahexaenoic acid
EFSA: European Food Safety Authority
ELISA: Enzyme-linked immunosorbent assay
EU: European Union
HPLC: High-performance liquid chromatography
ICP: Inductively coupled plasma
KIGGS: German Health Interview and Examination Survey for Children and Adolescents
LC/MS: Liquid chromatography-mass spectrometry
MMA: Methylmalonic acid
PUFA: Polyunsaturated fatty acid
RDI: Red cell distribution index
RDW: Red cell distribution width
T3: Triiodothyronine
T4: Thyroxin
TSH: Thyroid-stimulating hormone
VEBU: German Vegetarian Association
WHO: World Health Organization
